# Descriptions of three new species of the Termitophilous tribe Termitopaediini in China (Coleoptera, Staphylinidae, Aleocharinae)

**DOI:** 10.3897/zookeys.424.7670

**Published:** 2014-07-08

**Authors:** Xiao-Bin Song, Li-Zhen Li

**Affiliations:** 1 Department of Biology, College of Life and Environmental Sciences, Shanghai Normal University, 100 Guilin Road, Xuhui District, Shanghai 20023

**Keywords:** Termitopaediini, *Dioxeuta*, *Termitopulex*, termitophily, taxonomy, new species, China, Oriental region

## Abstract

Three new species belonging to two genera of the aleocharine tribe Termitopaediini Seevers, *viz.*, *Dioxeuta rara* Song & Li, **sp. n.**, *D. yunnanensis* Song & Li, **sp. n.**, and *Termitopulex sinensis* Song & Li, **sp. n.** from Baihualing Natural Reserve (Southwest China: Yunnan) are described and illustrated. This is the first record of *Termitopulex* Fauvel, 1899 from China.

## Introduction

The termitophilous tribe Termitopaediini Seevers was established by Seevers (1957) and subsequently revised by Kistner (1968, 2001). Up to date, 19 valid genera of the tribe have been known from the Oriental and Afrotropical regions (Kistner 2001). Pace (1999) described the first and only Chinese termitopaediine species, *Dioxeuta rougemonti* Pace, 1999 from Hong Kong. Recently, the senior author and his colleagues surveyed the termitophilous and myrmecophilous staphylinid fauna in the Baihualing Natural Reserve (Southwest China: Yunnan), and collected a series of unidentified aleocharine beetles from the fungus garden in a nest of termite *Macrotermes* Holmgren (Fig. 5A). A closer examination of this material revealed two new species of the genus *Dioxeuta* Sharp and one of the genus *Termitopulex* Fauvel, which are described herein.

## Material and methods

Holotypes and most of the paratypes are deposited in the Insect Collection of the Shanghai Normal University, Shanghai, China (SNUC), and some of paratypes are deposited in the Kyushu University Museum, Fukuoka, Japan (KUM).

Specimens were killed with ethyl acetate and preserved in 75% ethanol before dissection; photos of habitus were taken by a Canon EOS 7D with an MP-E 65mm macro photo lens; photos of characteristic pattern were taken by a Canon G9 Camera mounted on an Olympus CX31 microscope.

The following abbreviations are applied in the text: BL – body length, from the anterior margin of the head to the posterior margin of the abdominal tergite VIII; FBL – forebody length, from the clypeal anterior margin to the posterior margin of elytra; HL – head length, from the clypeal anterior margin to the occipital constriction; PL – length of the pronotum along the midline; HW – width of the head across the eyes; PW – maximum width of the pronotum.

## Taxonomy

### Dioxeuta

Taxon classificationAnimaliaColeopteraStaphylinidae

Sharp

Dioxeuta Sharp, 1899: 205 (original description, type species: *Dioxeuta microps* Sharp, 1899); Blackwelder 1952: 128 (discussion of type species); Seevers 1957: 217 (redescription, placed in tribe Termitopaediini); Kistner 1968: 169 (redescription, key to species); Kistner 2001: 17 (redescription; key to species).
Jacobsonella Silvestri, 1911: 59 (original description, type species: *Jacobsonella termitobia* Silvestri, 1911); Blackwelder 1952: 206 (discussion of type species); Seevers 1957: 217 (synonymized with *Dioxeuta*).

#### Remarks.

The genus is similar to *Neodioxeuta* Seevers and *Termitopulex* Fauvel by the shape of head and thorax. It can be easily separated from *Neodioxeuta* by the abdomen only slightly physogastric and the abdominal segment II represented by a reduced tergite only. It can be distinguished from *Termitopulex* by the outer paratergites about twice the width of the inner paratergites (Kistner 1968, 2001).

### Dioxeuta rara sp. n.

Taxon classificationAnimaliaColeopteraStaphylinidae

http://zoobank.org/FDB7E398-A593-4706-A236-2E551EC7E211

Fig. 1

#### Type material.

**Holotype: China:** ♂ (on slide), labeled ‘CHINA: Yunnan, Baoshan City, Mangkuan Town (芒宽乡), Baihualing N. R. (百花岭), 25°17'47"N, 98°48'22"E, alt. 1400 m, 23-IV-2013, Xiao-Bin Song leg. / HOLOTYPE [red], *Dioxeuta rara* sp. n., Song & Li det. 2014, SNUC’. **Paratype: China:** 1 ♀, same data as holotype, bearing the following label: ‘PARATYPE [yellow], *Dioxeuta rara* sp. n., Song & Li det. 2014, SNUC’.

#### Comparative notes.

*Dioxeuta rara* is most similar to *Dioxeuta rougemonti* and *Dioxeuta yunnanensis* described below through its yellowish-brown color and the presence of macrochaetae on abdominal tergite VII. The new species can be readily separated from *Dioxeuta rougemonti* by the deferent abdominal macrochaetotaxy as well as the shape of aedeagal median lobe. It differs from *Dioxeuta yunnanensis* by the presence of short macrosetae on abdominal tergites VII; tergite VIII with 5 pairs of macrosetae; sternite VIII almost truncate at apex and the deferent shapes of aedeagal median lobe and spermatheca.

#### Description.

**Figure 1.**
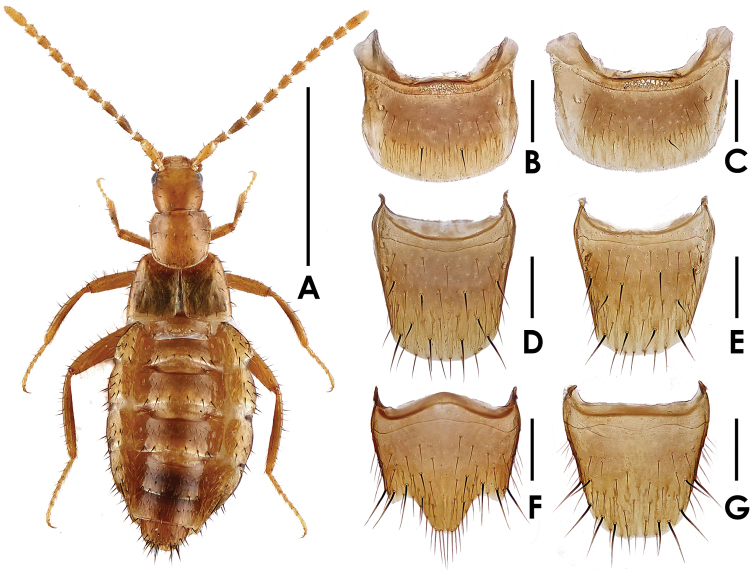
*Dioxeuta rara* sp. n. **A** holotype, female habitus **B** male tergite VII **C** female tergite VII **D** male tergite VIII **E** female tergite VIII **F** male sternite VIII **G** female sternite VIII. Scales (mm): **A** = 1; **B–G** = 0.2.

Body (Fig. 1A) shining, smooth. Coloration: fore body and legs yellowish-brown; antennae yellowish-brown to brown; abdomen yellowish-brown, with mesal area of tergites brown.

Head (Fig. 1A) subquadrate, slightly longer than wide, about 0.89 times as wide as long; sparsely covered with long setae. Pronotum (Fig. 1A) slightly wider than long, about 1.05 times as wide as long; with 7 pairs of macrosetae, with a row of 4 macrosetae along the anterior margin, 6 on disc and 2 each along the lateral margins. Elytra (Fig. 1A) wider than long; disc sparsely covered with macrosetae; hypomera with several long yellow setae along lateral margin. Abdomen slightly physogastric, widest at segments III–V; abdominal tergite VII shaped as in Figs 1B–C, sparsely covered with long yellow setae, posterior margin with a row of 8 very short setae; tergite VIII (Figs 1D–E) with 5 pairs of macrosetae. Macrochaetotaxy of abdominal tergites II–VIII: 4, 6, 6, 6, 6, 1–2 (short), 10; inner paratergites without macrosetae but sparsely covered with yellow setae; macrochaetotaxy of outer paratergites III–VII variable: 6–7, 5–7, 4–5, 2–3, 1.

**Figure 2.**
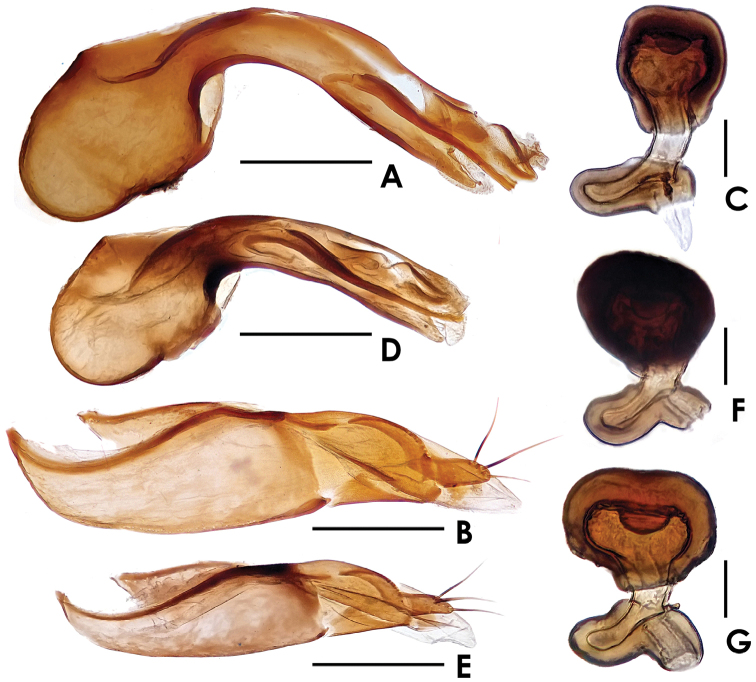
*Dioxeuta rara* sp. n. **A** median lobe of aedeagus, in lateral view **B** paramere **C** spermatheca *Dioxeuta yunnanensis* sp. n. **D** median lobe of aedeagus, in lateral view **E** paramere **F** spermatheca **G** ditto. Scales (mm): **A, B, D, E** = 0.2; **C, F, G** = 0.03.

Male. Sternite VIII almost truncate at apex, shaped as in Fig. 1F; with 5 pairs of macrosetae. Median lobe of aedeagus (Fig. 2A) with apical lobe distinctly curved; paramere shaped as in Fig. 2B.

Female. Sternite VIII slightly emarginate at apex, shaped as in Fig. 1G; with 5 pairs of macrosetae. Spermatheca shaped as in Fig. 2C.

#### Measurements.

**Male:** BL: 2.63; FBL: 1.06; HL: 0.38; HW: 0.35; PL: 0.41; PW: 0.45; HW/HL: 0.92; PW/PL: 1.05; HW/PW: 0.78. **Female:** BL: 2.65; FBL: 1.10; HL: 0.40; HW: 0.35; PL: 0.40; PW: 0.43; HW/HL: 0.88; PW/PL: 1.05; HW/PW: 0.81.

#### Distribution.

Southwest China: Yunnan.

#### Symbiotic host.

*Macrotermes* sp.

#### Etymology.

The Latin adjective *rāra* means ‘rare’.

### Dioxeuta yunnanensis sp. n.

Taxon classificationAnimaliaColeopteraStaphylinidae

http://zoobank.org/E47D43BF-D6B2-4A5F-A736-C172C63EB8C2

Fig. 3

#### Type material.

**Holotype: China:** ♂, labelled ‘CHINA: Yunnan, Baoshan City, Mangkuan Town (芒宽乡), Baihualing N. R. (百花岭), 25°17'47"N, 98°48'22"E, alt. 1400 m, 23-IV-2013, Xiao-Bin Song leg. / HOLOTYPE [red], *Dioxeuta yunnanensis* sp. n., Song & Li det. 2014, SNUC’. **Paratypes: China:** 1 ♂, 9♀♀, same data as holotype, bearing the following label: ‘PARATYPE [yellow], *Dioxeuta yunnanensis* sp. n., Song & Li det. 2014, SNUC’. (SNUC, KUM).

#### Comparative notes.

*Dioxeuta yunnanensis* is most similar to *Dioxeuta rara* described above, but can be readily separated from it by the different abdominal macrochaetotaxy as well as the shape of aedeagal median lobe and spermatheca.

#### Description.

**Figure 3.**
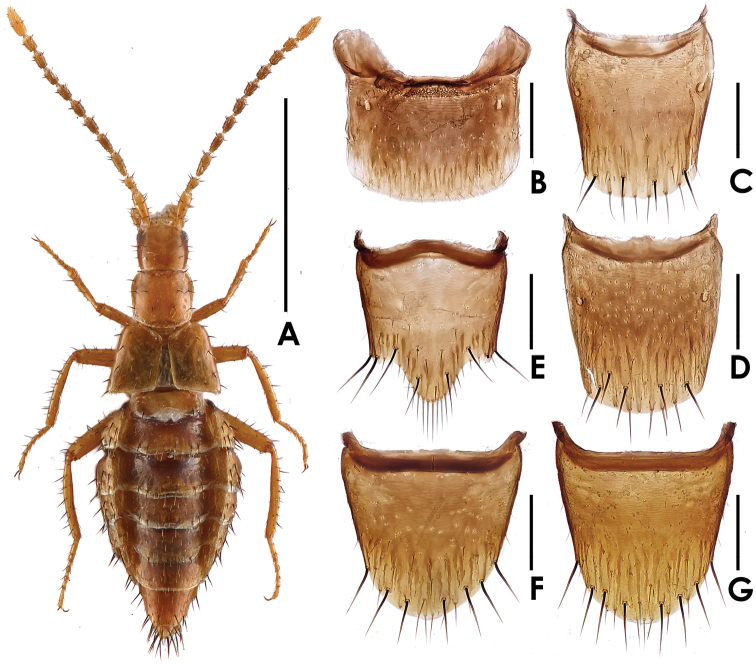
*Dioxeuta yunnanensis* sp. n. **A** male habitus **B** tergite VII **C** tergite VIII **D** ditto **E** male sternite VIII **F** female sternite VIII **G** ditto. Scales (mm): **A** = 1; **B–G** = 0.2.

Body (Figs 3A, 5B) shining, smooth. Coloration: fore body and legs yellowish-brown; antennae yellowish-brown to brown; abdomen yellowish-brown, with mesal area of tergites brown.

Head (Fig. 3A) subquadrate in form; slightly longer than wide, about 0.86 times as wide as long; sparsely covered with long setae. Pronotum (Fig. 3A) slightly wider than long, about 1.02 times as wide as long; with 7 pairs of macrosetae, with a row of 4 macrosetae along the anterior margin, 6 on disc and 2 each along the lateral margins. Elytra (Fig. 3A) wider than long; disc sparsely covered with macrosetae; hypomera with several short yellow setae along lateral margin. Abdomen slightly physogastric, widest at segments III–V; abdominal tergite VII shaped as in Figs 3B; tergite VIII (Figs 3C, E) with 4–5 macrosetae, posterior margin with a row of 6–8 long yellow setae. Macrochaetotaxy of abdominal tergites II–VIII: 2, 6, 6, 6, 4–6, 0, 4–5; inner paratergites without macrosetae but sparsely covered with yellow setae; macrochaetotaxy of outer paratergites III–VII is variable: 5–8, 4–6, 3–4, 0, 0.

Male. Sternite VIII rounded at apex, shaped as in Fig. 3D; with 4 pairs of macrosetae. Median lobe of aedeagus (Fig. 2D) with apical lobe curved; paramere shaped as in Fig. 2E.

Female. Posterior margin of sternite VIII (Fig. 3F–G) nearly rounded; with 7–8 macrosetae. Spermatheca shaped as in Fig. 2F–G.

#### Measurements.

**Male:** BL: 2.34–2.46; FBL: 0.95–1.06; HL: 0.35; HW: 0.30; PL: 0.38; PW: 0.38; HW/HL: 0.87; PW/PL: 1.00; HW/PW: 0.79. **Female:** BL: 2.61–2.72; FBL: 1.02–1.06; HL: 0.35–0.38; HW: 0.32; PL: 0.37–0.40; PW: 0.39–0.41; HW/HL: 0.84–0.87; PW/PL: 1.03–1.04; HW/PW: 0.78–0.82.

#### Distribution.

Southwest China: Yunnan.

#### Symbiotic host.

*Macrotermes* sp.

#### Etymology.

Named after its type locality of Yunnan Latinized.

### Termitopulex

Taxon classificationAnimaliaColeopteraStaphylinidae

Fauvel

Termitopulex Fauvel, 1899: 37 (original description, type species: *Termitopulex grandicornis* Fauvel, 1899); Blackwelder 1952: 379 (discussion of genotype); Seevers 1957: 222; (placed in tribe Termitopaediini); Kistner 1968: 153 (redescription; key to species).
Silvestrinus Bernhauer, 1932: 14 (original description, type species: *Silvestrinus erythraeanus* Bernhauer, 1932); Blackwelder 1952: 352 (discussion of the type species); Seevers 1957: 222 (synonymized with *Termitopulex*).

#### Remarks.

The genus is similar to *Polyteinia* Bernhauer and *Paratermitopulex* Kistner through the overall shape. It can be easily separated from *Polyteinia* by the absence of easily visible exit pores from abdominal segment VII and the different shape of paramere. It can be distinguished from *Paratermitopulex* by the rounded lateral edges of the pronotum. *Termitopulex* is also related to *Dioxeuta* from which it can be distinguished the less physogastric abdomen and the paratergites approximately equal in width (Kistner 2001).

### Termitopulex sinensis sp. n.

Taxon classificationAnimaliaColeopteraStaphylinidae

http://zoobank.org/3759E492-F1D6-4421-99DA-FCA7C212794D

Fig. 4

#### Type material.

**Holotype: China:** ♂, labelled ‘CHINA: Yunnan, Baoshan City, Mangkuan Town (芒宽乡), Baihualing N. R. (百花岭), 25°17'47"N, 98°48'22"E, alt. 1400 m, 23-IV-2013, Xiao-Bin Song leg. / HOLOTYPE [red], *Termitopulex sinensis* sp. n., Song & Li det. 2014, SNUC’. **Paratypes: China:** 3 ♂♂, 8♀♀, same data as holotype, bearing the following label: ‘PARATYPE [yellow], *Termitopulex sinensis* sp. n., Song & Li det. 2014, SNUC’. (SNUC, KUM).

#### Comparative notes.

*Termitopulex sinensis* can be readily separated from the only Asian congener *Termitopulex omaniensis* Kistner by the posterior margin of abdominal tergite VIII being broadly concave and the different abdominal macrochaetotaxy. The new species differs from other species of the genus by the different macrochaetotaxy of abdomen as well as the shape of aedeagus and spermatheca.

#### Description.

**Figure 4.**
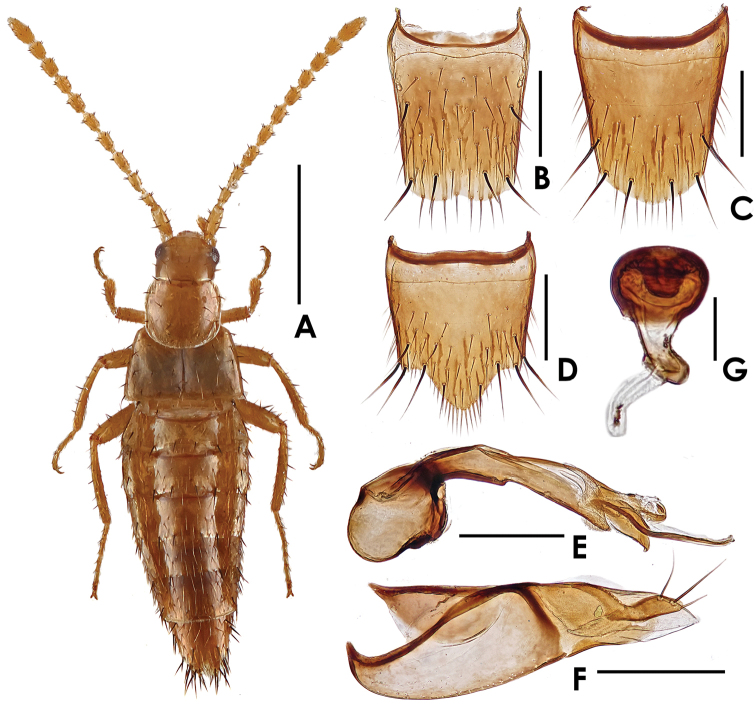
*Termitopulex sinensis* sp. n. **A** male habitus **B** tergite VIII **C** female sternite VIII **D** male sternite VIII **E** median lobe of aedeagus, in lateral view **F** paramere **G** spermatheca. Scales (mm): **A** = 1; **B–F** = 0.2; **G** = 0.03.

**Figure 5.**
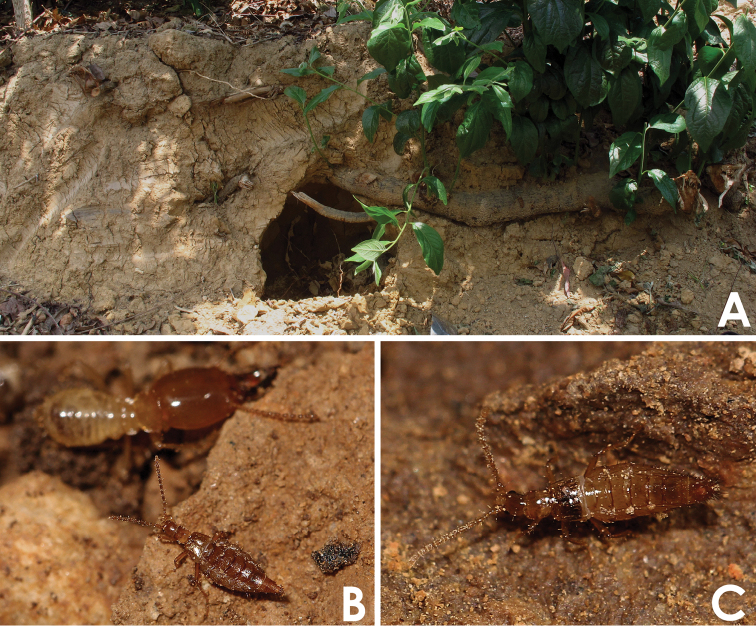
**A** over view of the symbiotic host’s nest **B** Living *Dioxeuta yunnanensis* **C** Living *Termitopulex sinensis*.

Body (Figs 4A, 5C) shining, smooth. Coloration: fore body and legs yellowish-brown; abdomen yellowish-brown, with tergites VI–VIII darker.

Head (Fig. 4A) subquadrate in form; slightly longer than wider, about 0.92 times as wide as long; with 4 macrosetae on disc and 1 each along the lateral margins. Mandibles with apical teeth elongate. Pronotum (Fig. 4A) slightly wider than long, about 1.06 times as wide as long; with 6 pairs of macrosetae, with a row of 4 macrosetae along the anterior margin, 4 on disc and 2 each along the lateral margins. Elytra (Fig. 4A) wider than long; disc sparsely covered with macrosetae. Abdomen widest at segments III–V; posterior margin of tergite VIII (Fig. 4B) broadly concave. Macrochaetotaxy of abdominal tergites II–VIII: 2, 4, 4, 4, 4, 2 (very short), 6; inner paratergites without macrosetae but covered with brown setae; macrochaetotaxy of outer paratergites III–VII as follow: 2, 1–2, 1–2, 0, 0.

Male. Sternite VIII rounded at apex; shaped as in Fig. 4D; with 6 pairs of macrosetae. Median lobe of aedeagus and paramere shaped as in Fig. 4E–F.

Female. Posterior margin of sternite VIII (Fig. 4C) nearly rounded; with 4 pairs of macrosetae. Spermatheca shaped as in Fig. 4G.

#### Measurements.

**Male:** BL: 2.08–2.31; FBL: 0.88–0.92; HL: 0.32; HW: 0.29; PL: 0.32–0.33; PW: 0.34–0.35; HW/HL: 0.92; PW/PL: 1.06–1.08; HW/PW: 0.85–0.88. **Female:** BL: 2.38–2.76; HL: 0.32–0.35; HW: 0.31–0.32; PL: 0.32–0.37; PW: 0.34–0.38; HW/HL: 0.89–0.96; PW/PL: 1.03–1.08; HW/PW: 0.82–0.84.

#### Distribution.

Southwest China: Yunnan.

#### Symbiotic host.

*Macrotermes* sp.

#### Etymology.

Named after the type locality.

## Supplementary Material

XML Treatment for Dioxeuta

XML Treatment for Dioxeuta rara

XML Treatment for Dioxeuta yunnanensis

XML Treatment for Termitopulex

XML Treatment for Termitopulex sinensis
